# Lethal Heat and Humidity Events

**DOI:** 10.1146/annurev-environ-111523-102139

**Published:** 2025-10-06

**Authors:** Shuang Zhou, Yao Wu, Yanming Liu, Yuan Gao, Pei Yu, Rongbin Xu, Gongbo Chen, Tingting Ye, Wenhua Yu, Juan Antonio Añel, Peng Bi, Angie Bone, Kristie L. Ebi, Antonio Gasparrini, Masahiro Hashizume, Ollie Jay, Yoonhee Kim, Sarah Perkins-Kirkpatrick, Jason Kai Wei Lee, Tiantian Li, Shao Lin, Lina Madaniyazi, Jinah Park, Fontina Petrakopoulou, Xerxes Seposo, Aurelio Tobias, Shanshan Li, Yuming Guo

**Affiliations:** 1 Climate, Air Quality Research (CARE) Unit, School of Public Health and Preventive Medicine, Monash University, Melbourne, Victoria, Australia; 2 EPhysLab, CIM-UVigo, Universidade de Vigo, Ourense, Spain; 3 School of Public Health, The University of Adelaide, Adelaide, South Australia, Australia; 4 School of Public Health and Preventive Medicine, Monash University, Melbourne, Victoria, Australia; 5 Center for Health and the Global Environment, University of Washington, Seattle, Washington, USA; 6 Centre for Statistical Methodology, Centre on Climate Change and Planetary Health, and Department of Public Health, Environments and Society, London School of Hygiene & Tropical Medicine, London, United Kingdom; 7 Department of Global Health Policy, Graduate School of Medicine, The University of Tokyo, Tokyo, Japan; 8 Heat and Health Research Incubator, Faculty of Medicine and Health, University of Sydney, Sydney, New South Wales, Australia; 9 Department of Global Environmental Health, Graduate School of Medicine, The University of Tokyo, Tokyo, Japan; 10 ARC Centre of Excellence for the Weather of the 21st Century, the Australian National University, Canberra, ACT, Australia; 11 Fenner School of Environment and Society, the Australian National University, Canberra, ACT, Australia; 12 Heat Resilience and Performance Centre, Yong Loo Lin School of Medicine, National University of Singapore, Singapore; 13 Human Potential Translational Research Program, Yong Loo Lin School of Medicine, National University of Singapore, Singapore; 14 China CDC Key Laboratory of Environment and Population Health, National Institute of Environmental Health, Chinese Center for Disease Control and Prevention, Beijing, China; 15 Departments of Epidemiology and Biostatistics and of Environmental Health Sciences, University at Albany, State University of New York, Rensselaer, New York, USA; 16 School of Tropical Medicine and Global Health, Nagasaki University, Nagasaki, Japan; 17 Department of Public Health Sciences, Graduate School of Public Health, Seoul National University, Seoul, Republic of Korea; 18 Energy Engineering and Climate Protection, Technische Universität Berlin, Berlin, Germany; 19 Institute of Environmental Assessment and Water Research (IDAEA), Spanish Council for Scientific Research (CSIC), Barcelona, Spain

**Keywords:** heat, humidity, health effect, heat health action plans, strategy

## Abstract

Climate change increasingly threatens global health as more frequent extreme heat events, combined with varying humidity levels, exacerbate both direct and indirect health risks, strain energy resources, and lead to economic loss. Vulnerable populations, including the elderly, young children, and those with preexisting health conditions, face greater risks due to lower physiological adaptive capacity. Those from socioeconomically disadvantaged communities are also vulnerable because of increased exposure and reduced capacity. While research has expanded our understanding of the physiological effects of extreme heat and humidity, challenges persist, including inconsistent data, lack of unified heat wave definitions, and limited knowledge of their impact on mortality and morbidity especially in specific populations. Addressing these challenges requires enhanced data and a comprehensive evaluation of humidity’s modifying effects. Global collaboration to strengthen heat health action plans is essential, with future efforts focusing on enhancing the accessibility and effectiveness of interventions, especially in underresourced regions.

## Introduction

Over the past decades, increasingly frequent and extreme heat events have characterized the era of climate change. Notable examples include the Chicago heat wave of 1995; the Central European heat wave of 2003; the Europe-Russia heat wave of 2010; the Pacific Northwest heat wave of 2021; and the unprecedented heat waves across Europe, North Africa, the Middle East, and China in 2022 and 2023 (1). Extreme heat events are intensifying worldwide, with hotspots emerging in regions such as the Middle East, eastern South America, and northern Africa, where the intensity, frequency, and duration of heat events are increasing at the fastest rates (2). These events have caused significant mortality and widespread health impacts (3, 4). In response, governments and international organizations have developed various heat health action plans (HHAPs), including heat health warning systems (HHWSs) (5, 6). However, current risk-reduction strategies remain inadequate. Traditional evaluation metrics in HHAP and HHWS designs often fail to account for inequalities in health risks across gender, age, and socioeconomic status, potentially limiting the effectiveness of universal risk-reduction strategies in both academic research and policymaking (7). Furthermore, while the physiological and biophysical effects of humidity in heat-related health outcomes are well-understood (8), their modified impact on heat-related health outcomes, particularly in vulnerable populations, remains unclear and is often overlooked in current risk-reduction strategies. Therefore, a targeted literature review is essential to summarize the current understanding of humid heat event risks, the vulnerability of specific populations, and the gaps in current risk-reduction strategies.

**Heat health action plans (HHAPs):** comprehensive public health strategies to mitigate the health risks associated with extreme heat events, integrating various measures to enhance community resilience and protect vulnerable populations during excessive heat

**Heat health warning systems (HHWSs):** integrated frameworks combining meteorological data with public health strategies that alert communities and decision-makers about impending extreme heat events, thereby mitigating associated health risks and enhancing preparedness

In this review, we first explore the spatiotemporal variations of historical extreme heat events since 1990. We then investigate recent epidemiological evidence regarding the effects of extreme heat events, with special attention to vulnerability and humidity. We also analyze the development and implementation of HHAPs and HHWSs across the world, focusing on identifying inequities in these strategies. Finally, we propose targeted strategies and potential improvements to mitigate the impacts of extreme heat events.

This work provides crucial insights into the occurrence, effects, and management of extreme heat events. These insights offer valuable guidance for mitigating the impacts of extreme heat and enhancing public health preparedness.

## Spatiotemporal Distribution Of Extreme Heat

### Definitions and Global Distributions of Extreme Heat

Extreme heat events are characterized by both high temperatures and prolonged durations, and they are typically classified as heat waves if they last for at least two or three consecutive days. The Intergovernmental Panel on Climate Change describes a heat wave as a period of abnormally and uncomfortably hot weather. However, there is no unified definition of a heat wave, with different criteria used in the literature. The climate science literature presents a plethora of criteria based on different temperature indicators (e.g., mean, minimum, or maximum temperatures), thresholds (e.g., relative measures such as the ninety-fifth percentile), and minimum durations (e.g., at least two or three consecutive days) (9). From a health protection perspective, an ideal threshold should effectively detect potentially harmful occurrences while avoiding overly frequent alerts that may lead to public fatigue and resource wastage (10). Traditionally, the threshold was determined based on mortality rates. However, recent studies indicate that more immediate metrics, such as emergency visits or ambulance service uses, might better reflect current risks and improve the responsiveness of HHWSs (11, 12). Extensive efforts have been made toward identifying the suitable threshold. Researchers have attempted to achieve this by incorporating advanced techniques such as machine learning; however, the most suitable threshold remains undetermined (13).

Defining heat waves based solely on temperature is a straightforward and easy-to-adopt approach. However, this method fails to capture the full extent of heat stress and does not reflect physiological responses to heat exposure and vulnerability (14). Ambient temperature is typically measured in the shade, which may not accurately represent actual exposure or net heat stress. Heat stress is influenced not only by air temperature but also by humidity, mean radiant temperature, and wind velocity. Wet-bulb globe temperature (WBGT), which integrates temperature, humidity, wind speed, and solar radiation, may serve as a more accurate indicator for defining heat waves, as it better reflects the environmental conditions that lead to heat stress (15). Therefore, to illustrate the distribution and trends of heat waves with and without considering humidity, we compared definitions using daily mean ambient temperature and WBGT on a global scale. We defined heat waves as when the temperature indicator exceeds the ninety-fifth percentile of the year-round frequency distribution for at least two consecutive days (12) from 1990 to 2023 (see the **Supplemental Methods**).

**Heat stress:** the perceived discomfort and physiological strain associated with exposure to a hot environment

**Figure 1:**
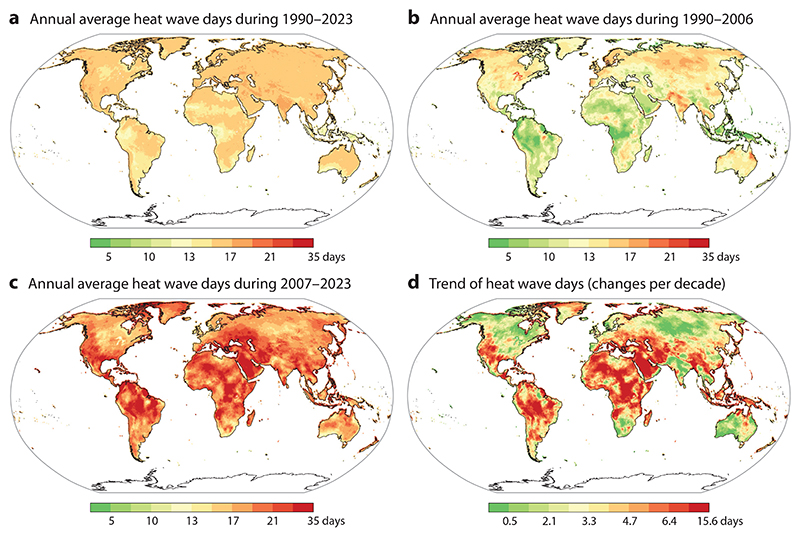
Maps of annual average heat wave days during 1990–2023 (*a*), in the first (*b*) and second (*c*) 17 years of 1990–2023, and the estimated trend during the period (*d*). The trend in heat wave days from 1990 to 2023 was determined using Sen’s slope estimator applied to the annual heat wave days during this period. Heat wave days in each grid cell was defined as a period of at least two consecutive days where daily mean ambient temperature exceeded the 95th percentile of the year-round frequency distribution for that cell.

Between 1990 and 2023, the mean annual number of heat wave days across all grid cells was 15.6 days (Figure 1a). During the first 17 years (1990–2006), the mean annual number of heat wave days was lower, averaging 12.0 days (Figure 1b). However, in the subsequent period (2007–2023), heat wave intensity increased, with the mean annual number of heat wave days rising to 19.3 days (Figure 1c). A clear global increase in heat wave days was observed, especially in Africa, the Middle East, and parts of Asia (Figure 1d).

**Figure 2:**
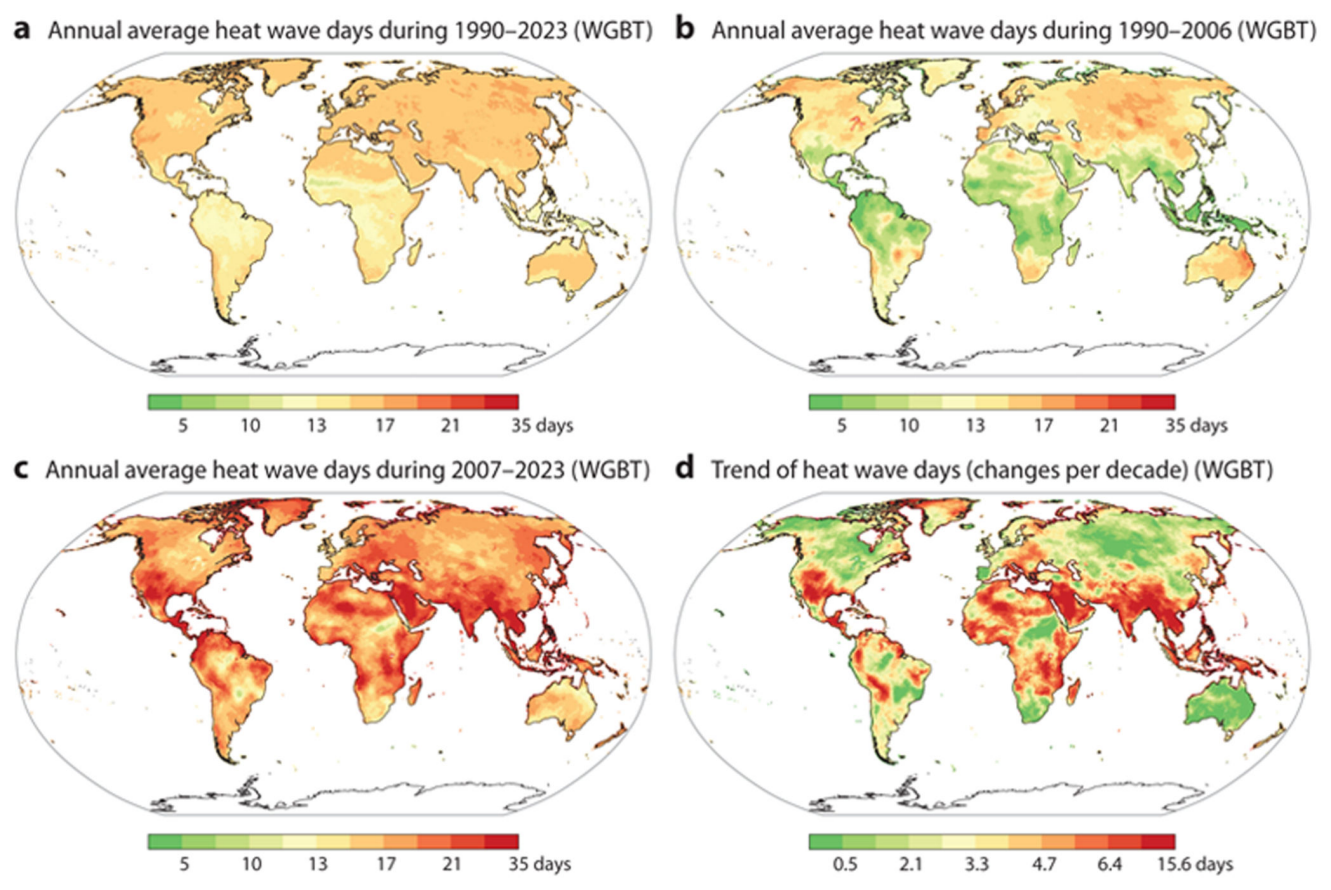
Maps of annual average heat wave days estimated using the wet globe bulb temperature (WGBT) during 1990–2023 (*a*), in the first (*b*) and second (*c*) 17 years of 1990–2023, and the estimated trend during the period (*d*). The trend in heat wave days from 1990 to 2023 was determined using Sen’s slope estimator applied to the annual heat wave days during this period. Heat wave days in each grid cell was defined as a period of at least two consecutive days where daily mean WGBT exceeded the 95th percentile of the year-round frequency distribution for that cell.

When using WBGT, the overall spatiotemporal patterns of heat waves were similar to those based on daily mean ambient temperature. From 1990 to 2023, the mean annual number of heat wave days across all grid cells was 15.5 days, increasing from 12.1 days during 1990–2006 to 18.8 days during 2007–2023 (Figure 2a–c). Overall, heat wave days defined by daily mean ambient temperature showed a more pronounced increase compared to WBGT-based estimates (Figure 2d).

### Future Projections of Extreme Heat

Projections from physical climate models are analyzed to understand probable future changes in heat waves. Global climate models (GCMs), such as those participating in the Coupled Model Intercomparison Project Phase 6, are a common tool employed by the climate community to understand broad-scale changes in heat waves under various future emission scenarios. Regional climate models that dynamically downscale GCMs can be used to understand how heat waves may develop and change over smaller spatial scales where impacts are experienced, noting that GCMs with a spatial resolution of 100 km or more are required for a global assessment. Under intensified human influence on the climate, increasing heat wave trends are expected to occur across the world (16, 17). Projected increasing regionally averaged heat wave characteristics scale with global temperature (16, 18); however, the strength of this correlation varies by location. For example, in southern Europe, the intensity of regionally averaged heat wave days is projected to double per degree of global warming, whereas in Australia, it is expected to increase by around 1.2°C per degree of global warming (16). Therefore, the precise relationship between global warming and heat waves may be stronger or weaker than described here, depending on the specific locations. At smaller spatial scales, heat waves are also influenced by factors beyond climate, such as land use (19), local geography (20), and population demographics (21). Moreover, projecting the occurrence and severity of record-breaking heat waves that infer adverse health impacts presents a significant challenge for GCMs. This difficulty arises because local-scale antecedent conditions and meteorology, crucial factors in these projections, are often not adequately captured in model simulations that span large domains and multiple decades (22). This limitation inhibits the development of an in-depth understanding of the future health risks posed by projected individual heat wave events directly from large-scale physical climate models at the spatial and temporal scales where impacts are most likely to be experienced. Therefore, assessing how heat inflicts adverse health impacts at local and city scales requires specific and ad-hoc tools, such as MesoNH, which can further model local-scale mechanisms such as urban heat islands.

Recently, more projections of heat stress have been yielded from physical climate models by incorporating humidity, benefiting from the recent improvements in data quality and availability (23). Under a high emissions pathway, half of the world’s population, predominantly residing in tropical and subtropical climates, could experience present-day WBGT thresholds that are 100 to 250 times more frequent by 2080 (24). Similarly, dangerous heat index values are projected to increase by 50–100% over tropical regions, and by three to ten times across midlatitude regions (25), along with dramatic increases in heat-related mortality attributable to global warming (26). Under an emissions-intensive future, regions such as the Middle East may become uninhabitable due to the increased frequency of deadly extreme heat events (27). Moreover, there is a rapidly growing body of evidence indicating that many regions are expecting increasing trends in the frequency and severity of adverse heat stress closely linked to intensified anthropogenic influence on the climate. Notably, many locations susceptible to future dangerous heat stress are also densely populated and thus more vulnerable (28, 29).

## Effect Of Extreme Heat And Humidity

**Figure 3:**
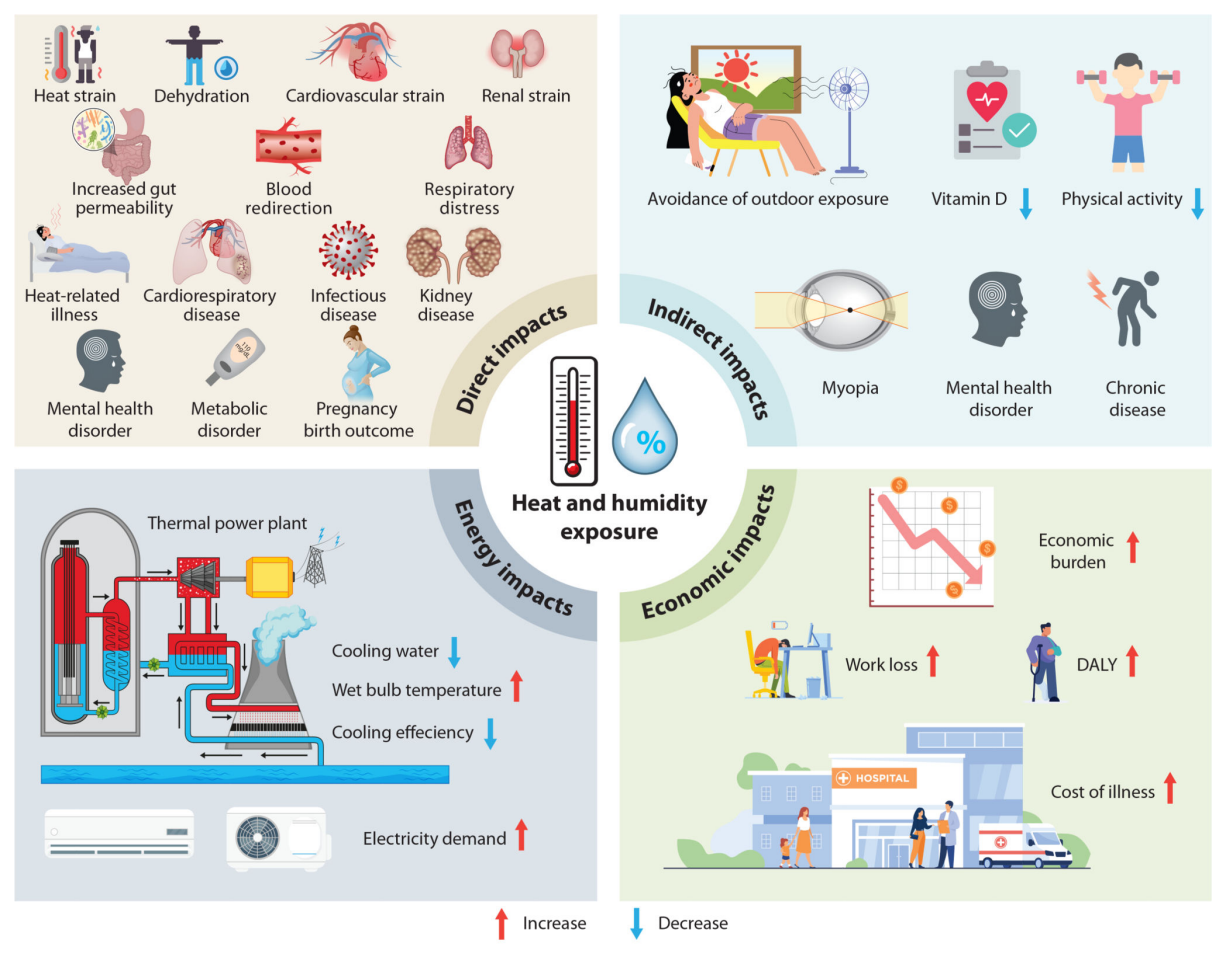
Impacts of extreme heat and humidity and their mechanisms. This figure illustrates the interconnected pathways through which heat and humidity exposure affect human health and societal systems. Direct health impacts include heat strain, dehydration, cardiovascular and renal strain, and respiratory distress. High humidity impairs sweat evaporation, elevating core temperature, altering blood flow, and compromising organ function. These physiological stresses increase the risk of cardiorespiratory, metabolic, infectious, and mental health disorders, as well as adverse pregnancy outcomes. Indirect impacts arise from behavioral adaptations such as reduced outdoor activity, leading to vitamin D deficiency and physical inactivity, and increased risks of chronic diseases, myopia, and mental health issues. Energy impacts stem from reduced cooling efficiency and water availability in thermal power plants, along with increased electricity demand. Economic impacts include increased healthcare costs, work productivity losses, and rising DALYs, leading to a growing economic burden. Abbreviation: DALY, disability-adjusted life year. One DALY represents the loss of the equivalent of one year of full health. DALYs for a disease or health condition are the sum of the years of life lost due to premature mortality and the years lived with a disability due to prevalent cases of the disease or health condition in a population.

Extreme heat poses significant risks to human health, leading to a range of health issues, including heat-related illnesses, cardiorespiratory diseases, infectious diseases, kidney disorders, metabolic diseases, mental health disorders, and adverse pregnancy and birth outcomes. In addition, extreme heat strains energy production and consumption, resulting in economic loss and further health impacts (Figure 3).

**Heat-related illnesses:** a spectrum of mild to life-threatening health conditions that occur when the body is exposed to excessive heat and cannot adequately cool itself, ranging from dehydration to heat stroke

### Health Effects of Extreme Heat

Epidemiological studies across the globe have demonstrated a clear link between extreme heat and increased mortality (30). Research from Europe, the United States, and Asia reveals that during extreme heat events, mortality rates often surge, particularly from cardiovascular and respiratory conditions. For example, the 2003 European heat wave claimed nearly 70,000 lives (31).

Here, we summarize the epidemiological evidence on the health effects of extreme heat events, including extreme high temperatures, heat wave days, and notable extreme heat events in the Emergency Events Database (EM-DAT). We also investigate the disproportionate impact of these heat events on vulnerable populations and the modifying role of humidity (for a summary of health effects, see Supplemental Table 1).

#### Heat-related illness

Heat-related illness is a key indicator of extreme heat, ranging from mild conditions like heat rash to severe cases such as heat stroke. Heat stress occurs when elevated environmental temperatures cause the body to undergo thermoregulatory, acute-phase, and heat-shock responses, potentially leading to heat-related illness if not appropriately managed (32). Heat exhaustion is marked by symptoms such as nausea, vomiting, and muscle cramps due to fluid and salt loss and can progress to organ failure in severe cases (33). Heat stroke occurs when the core body temperature exceeds 40°C, and is characterized by symptoms such as hot, dry skin and severe neurological disturbances, including agitation and coma (32). Despite its severity, heat stroke is often under-reported due to misdiagnosis or delayed medical response (33). Although fatalities from heat-related illnesses are relatively rare, mortality rates can spike during heat waves. For example, the 2003 heat wave in France caused 1,670 deaths from heat stroke or hyperthermia and 1,754 from dehydration, out of a total of 15,000 excess deaths (34). A recent meta-analysis combining data from 30 studies found that for a 1°C increase in temperature above the study-specific baseline, morbidity and mortality from direct heat-related illness rose by 18% and 35%, respectively (35). The most significant increase in morbidity was reported for direct heat illness, followed by dehydration (35).

#### Cardiorespiratory diseases

Evidence shows that extreme heat significantly increases the risks of morbidity and mortality from cardiorespiratory diseases (36). A comprehensive meta-analysis involving 54 studies from 20 countries further reported that extreme heat enhanced the risk of cardiovascular and respiratory mortality by 14.9% and 18.3%, respectively (37). Region-specific studies in China have detailed how extreme heat amplifies risks across a spectrum of cardiovascular conditions, showing increases of 27.8% for total circulatory diseases and 26.7% for cerebrovascular disease, and varying degrees of increases for different forms of ischemic heart disease ranging from 25.2% to 32.2% (38). Physiological changes during extreme heat have been extensively explored, revealing significant associations with key clinical indicators such as heart rate and markers of inflammation and cellular stress, including neutrophil count, hematocrit, and lactate dehydrogenase. These findings highlight the systemic impact of heat on cardiovascular health (39).

#### Infectious diseases

Evidence regarding the impact of extreme heat on infectious diseases remains relatively sparse and inconsistent. Studies focusing on general infectious and parasitic diseases have consistently found increased risks during extreme heat (40, 41). However, specific diseases such as pneumonia showed inconsistent results: Chen et. al. (42) reported no significant change in risk while others indicated increased emergency department (ED) and hospital admissions (41, 43). Vector-borne diseases such as dengue exhibited both increases and decreases in incidence (44, 45), suggesting that local environmental and climatic conditions play crucial roles in disease transmission during extreme heat. Additionally, food-borne illnesses, particularly salmonellosis, were reported to increase during extreme heat, likely due to the enhanced growth of pathogens (46). Gastrointestinal infections such as cholera also saw increases during extreme heat, as observed in Bangladesh, highlighting the vulnerability of certain regions to heat-induced infectious outbreaks (47).

#### Kidney diseases

Extreme heat significantly influences the incidence of both acute kidney injuries (AKIs) and chronic kidney disease (CKD). AKIs, often triggered by extreme heat through mechanisms such as rhabdomyolysis and inflammation (48), can escalate into recurrent episodes that eventually lead to CKD and potentially kidney failure (49). A study in Adelaide, Australia, showed that daily minimum temperature was associated with an increase in daily ED admissions for total renal disease and specific renal disease (e.g., AKIs, renal failure, CKD, urolithiasis, and urinary tract infections) (49). Similar findings were also found in various climate zones such as China (50), Spain (51), New Zealand (52), and New York (53). A meta-analysis combining data from 82 studies confirmed such findings and found that with a 1°C increase in temperature, the risk of kidney-related morbidity increased by 1%, with the greatest risk for urolithiasis (54).

Apart from direct impacts on renal health, extreme heat also leads to emerging and reemerging vector-borne diseases such as dengue. These conditions can lead to glomerulonephritis, kidney tubular injury, and potentially severe AKIs that can progress to CKD (55).

#### Mental and behavioral disorders

Extreme heat could also increase the risks of mental and behavioral disorders (MBDs) and suicide or self-harm (56–59). However, the certainty of the evidence is low due to the insufficient number of studies and varying results from different definitions of heat waves. A meta-analysis reported that extreme heat increased the risk of MBD morbidity by 5.0% across seven studies compared with nonextreme-heat periods (56). Another meta-analysis involving three studies reported a 9.7% increase in hospital attendance or admissions for mental illness during extreme heat (58). The risks of mortality due to MBD and suicide for extreme heat were higher compared with morbidity. A meta-analysis reported that the risk of MBD mortality increased by 3.1% per 1°C increase in temperature, while the risk of MBD morbidity increased by 0.7% per 1°C increase in temperature (59). Similarly, the risk of completed suicide increased by 5.2%, while the risk of suicide attempts showed a smaller increase of 1.4% per 1°C increase in temperature (60). However, the risks of MBD mortality were heterogeneous. A comparative study between two cities—Rome, Italy, and Stockholm, Sweden—reported an increased risk of mortality due to psychiatric disorders in both cities during extreme heat (61). A similar risk was also found in Adelaide (62) but not in New South Wales (63) or Brisbane, Australia (64). Similarly, a multicountry study across 60 countries demonstrated correlations between extreme heat and suicide (65). Only 8 of the 60 countries showed significant results, with 3 showing an increased risk and 5 showing a decreased risk. Nevertheless, a recent study investigating the risk of mortality during the 2021 western North American heat dome in Canada demonstrated that the increased risk of mortality from extreme heat events was greater among individuals with comorbid psychiatric conditions, such as schizophrenia and substance use disorder, compared with those with other chronic diseases (66). Additionally, extreme heat has been associated with increased stress levels, which may contribute to higher rates of family and intimate partner violence (67).

#### Metabolic diseases

Evidence consistently demonstrates that extreme heat significantly exacerbates the risks of morbidity and mortality from metabolic diseases (68). For example, during a severe heat wave in July 2007 in Belgrade, Serbia, there was an excess mortality rate of 38%, with diabetes-related deaths increasing by 286% (69). Similarly, during the 2006 California heat wave, there were 16,166 excess ED visits and 1,182 additional hospitalizations, prominently featuring diabetes as one of the most affected conditions (70). Further research across 48 provinces in Spain found that extreme heat had a substantial impact on metabolic disorders, yielding a risk ratio of 1.98 for these conditions during extreme heat periods (71). Complementary findings from a prospective cohort study in China showed significant correlations between elevated temperatures and critical metabolic indicators such as blood lipids, uric acid, and fasting plasma glucose (72). Additionally, a literature review summarized that the overall pooled effect of extreme heat on diabetes among older adults resulted in a risk ratio of 1.10 (68). This effect was particularly evident during transitional months, with a case-crossover study in New York State indicating that each interquartile rise in temperature was associated with a notable increase in diabetes hospitalizations, primarily occurring in the month of May but not during the peak summer months (73).

#### Adverse pregnancy and birth outcomes

Pregnant women are particularly susceptible to the effects of heat waves, which can result in a range of adverse pregnancy and birth outcomes. Exposure to high temperatures has been associated with increased risks of maternal stress (74), gestational diabetes mellitus (GDM), and hypertensive disorders of pregnancy (HDP). The second trimester is notably crucial for the development of GDM (75), while the onset and end of pregnancy are critical periods for HDP (76).

The risks of miscarriage or spontaneous abortion are also associated with extreme heat, with the third trimester being particularly vulnerable (77). This stage is crucial as heat waves can impair fetal growth (78), increasing the risk of low birth weight and abnormal size for gestational age (including both small for gestational age and large for gestational age) (79), as well as preterm birth (80).

Moreover, extreme heat has been implicated in an increased risk of congenital heart defects, with the most critical period occurring during weeks 2–8 of gestation (81). The risks extend to stillbirths and infant mortality, particularly when extreme heat exposure occurs close to the time of delivery. A study in the United States indicated that a 1°C increase in temperature during the week before delivery, relative to seasonal norms, could raise stillbirth risks by 6%, which translates to an additional 4 stillbirths per 10,000 births (82).

#### Disproportionate effect on vulnerable populations

Extreme heat presents significant risks to all populations, but certain groups are disproportionately affected according to both physiological factors, such as age, sex, and health status, and exposure factors such as occupation and socioeconomic conditions. The elderly, particularly those over 65, are highly vulnerable during extreme heat (36, 37); for example, individuals aged over 75 accounted for 90% of all excess deaths during the 2007 heat wave in Serbia (69). This vulnerability is largely due to elevated cardiovascular strain induced by heat stress, which is a predominant cause of death among older adults during extreme heat (83). Young children (under 5 years old) and infants also face heightened risks due to less efficient thermoregulatory systems and their greater reliance on adults for cooling and protection. Reports from the 2006 California heat wave indicated a sharp increase in ED visits among young children, underscoring the acute risks to this group (70). Sex also modifies the associations. During the 2007 heat wave in Serbia, the excess mortality in females (54%) was over two times higher than that in males (23%) (69). Females are reported to have higher risks of heat wave–related cardiovascular disease, pneumonia (43), and diabetes (84) than males. Conversely, studies showed that the risk of suicide associated with extreme heat is higher among males than females (60, 85). People with preexisting conditions, especially those affecting the cardiovascular, respiratory, or renal systems, are at higher risk because the heat places additional stress on these already compromised systems (84, 86). Occupational exposure further exacerbates these disparities, with outdoor workers in fields such as agriculture or construction facing higher risks due to direct and prolonged exposure to heat and dehydration (87). Additionally, residents of low- and middle-income countries suffer extreme heat disproportionately due to inadequate healthcare infrastructure and limited resources for heat adaptation, further exacerbating the health impacts of extreme heat on these populations (36).

#### Modifying role of humidity

The role of humidity in exacerbating extreme heat effects is significant and complex (88). High humidity not only increases the perceived temperature but also impairs the body’s capacity to dissipate heat through suppressed sweat evaporation, amplifying the physiological challenges and related health risks (8, 89). This interaction highlights the importance of employing comprehensive heat indices, such as apparent temperature, WBGT, and humidex, to accurately assess heat stress and its related health effects. Current research has shown that these indicators, along with the heat wave events they define, are associated with more severe health outcomes (90–92). However, none of these indicators is consistently superior to other indices or to dry-bulb temperature in predicting population-level heat-related mortality impacts (93, 94). The optimal predictor varied across different age groups, seasons, and cities (93, 94).

**Apparent temperature:** the temperature equivalent perceived by humans, caused by the combined effects of air temperature, relative humidity, and wind speed

**Humidex:** a Canadian-developed index that combines air temperature and humidity to reflect how hot it feels to the average person during warm, humid conditions; similar to the heat index used in other countries

Moreover, epidemiological evidence regarding the modifying effect of humidity is inconsistent. Some studies indicate that high temperature and high humidity increase the mortality rate of cardiovascular disease (95) and metabolic syndromes (96). In contrast, others suggest that low temperature combined with high humidity is a high-risk factor for mortality from cardiovascular disease (97–99). Research from the Pennsylvania State University Human Environmental Age Thresholds Project indicates that even 34°C in humid conditions can substantially increase heart rate, intensifying cardiovascular strain (100). In contrast, other investigations highlight scenarios where lower humidity levels might significantly alter the impacts of extreme heat on mortality (41) and conditions such as acute bronchitis and bronchiolitis (71). Furthermore, recent physiological models show that survivability thresholds in humid conditions vary significantly based on factors such as age and sun exposure. Older adults, in particular, have lower tolerances to heat and humidity, highlighting their increased vulnerability compared to younger adults (101). Current evidence emphasizes the complex and varied nature of humidity’s role in heat-related health dynamics, necessitating tailored approaches in public health planning and response strategies to effectively mitigate these risks.

#### Mechanisms for health impact of extreme heat modified by humidity

The synergistic relationship between extreme heat and humidity precipitates a complex array of physiological consequences. The direct physiological mechanisms encompass heat strain, dehydration, cardiovascular strain, renal strain, and respiratory distress, potentially culminating in heat exhaustion, heat stroke, and an elevated risk in cardiovascular and respiratory morbidities and mortalities. High humidity impedes sweat evaporation, compromising the body’s ability to cool itself, thereby intensifying the risk of heat strain, dehydration, and cardiovascular strain. Elevated body temperatures cause blood to be redirected from the core to the skin to aid in heat dissipation. This decrease in splanchnic blood flow, along with high internal temperature, can increase gut permeability, allowing bacteria to enter the bloodstream (102). As a result, widespread clotting and multiple organ failure can occur, which may be fatal. Individuals with cardiovascular disease are at a high risk for heat-related illnesses, not necessarily due to overheating but because of a reduced ability to compensate for increased cardiovascular strain during heat exposure (103, 104). This strain is caused by the need for higher cardiac output to maintain blood pressure in the face of profound levels of cutaneous vasodilation, which can lead to cardiovascular collapse in people with an underlying cardiovascular infirmity (4). Dehydration further worsens this risk by decreasing blood volume, which reduces stroke volume and requires a higher heart rate to maintain cardiac output. People with preexisting kidney disease are at higher risk of renal failure during exposure to extreme heat and humidity, primarily due to reduced blood flow to the kidneys, which can cause low oxygen delivery and acute injury to hypoxia-sensitive areas (105). Chronic dehydration can also lead to kidney fibrosis and CKD.

Slow and gradual increases in average temperatures can cause significant negative effects on systems ranging from infrastructure to the human body (106). One of the indirect consequences of extreme heat (such as in the tropics) is chronic avoidance of outdoor exposure (107), precipitating vitamin D deficiency, and physical inactivity, which would incur negative physical, mental, and even eye health issues. For example, increased outdoor activity during late adolescence and young adulthood reduces the risk of developing late-onset myopia (≥15 years of age) (108). The diminution of outdoor activity, motivated by fear of heat-related illnesses, contributes to a sedentary lifestyle, increasing the risk of chronic diseases, including obesity, diabetes, and cardiovascular disease. Moreover, social isolation and reduced outdoor engagement can precipitate depression, anxiety, and other mental health disorders. The health impacts of heat waves, modified by humidity, necessitate a nuanced understanding of the direct and indirect impacts.

### Energy Production and Use

Access to adequate technology to adapt to environmental conditions and reduce the health impacts of extreme heat is of the utmost importance. However, extreme heat significantly affects power production, transmission, and consumption (109, 110). For instance, the heat waves in Europe in 2003 highlighted the challenges faced by hydropower and thermal power plants (TPPs). These events reduced water availability for cooling, impairing hydropower output and decreasing TPP efficiency (111, 112). Additionally, heat waves often cause disruptions in hospital operations due to power supply shortages or excessive demand for electricity (113). Over the past 20 years, the interplay between heat, humidity, and water resources has underscored the critical connection to global electricity generation (114, 115). However, the effects of compound extreme weather events, such as high temperatures combined with high humidity, on the energy sector remain largely unexplored.

Rising global temperatures and altered humidity patterns significantly affect water availability, which is essential for electricity generation. Hydropower, in particular, is highly vulnerable to climate variability due to its direct dependance on river flows and reservoir levels, which are influenced by droughts, heavy rainfall, and shifts in precipitation patterns. During drought conditions, reduced water availability limits cooling processes for TPPs, further impairing thermal efficiency and hydropower output. Moreover, shifts in the timing and intensity of precipitation reduce the predictability of water flows, complicating hydropower resource management.

A case study has demonstrated that wet-bulb temperature (WBT) is a key variable influencing the efficiency of combined cycle TPPs (116). When WBT exceeds 20°C, the power plant efficiency declines by approximately 1% for each additional degree Celsius. Humid heat waves, in particular, pose a significant threat to the energy sector in subtropical Asia, especially in countries such as India and China, where roughly 70% of the electricity is generated from TPPs (117, 118).

**Wet-bulb temperature (WBT):** the lowest temperature to which air can be cooled by the evaporation of water into the air at a constant pressure

Meanwhile, humidity during extreme heat plays a critical role in driving electricity demand (119). For example, during the summer, WBT is the primary factor explaining electricity demand in the United States (120), with an increase in demand of 50% when humidity is considered, compared to models that exclude it. By 2050, air conditioning demand in urban areas is expected to rise by up to 75% due to increasing WBT with climate change (121).

To mitigate the impact of humid heat waves on water resources and electricity generation, several strategies are essential, including improving water-use efficiency, developing advanced water management technologies, and diversifying energy sources (122). Integrating renewable energy, such as wind and solar, can also reduce dependance on both hydropower and TPPs (123).

### Economic Effect

Extreme heat exposure significantly impacts healthcare costs, including ED and hospital admissions. Epidemiological analysis in Sydney, Australia’s largest city, revealed that from 2010 to 2016, the total health service costs attributed to extreme heat exposure amounted to approximately A$252 million. The largest expenses arose from mental health hospital admissions, followed by admissions for ischemic heart and renal diseases. These costs are expected to rise markedly to A$387–399 million by the 2030s and A$506–570 million by the 2050s under various climate change scenarios (124). In the United States, the 2006 California heat wave led to an estimated $14 million in medical costs for all-cause excess ED visits (125). In China, costs of approximately 0.18 billion CNY (about US$26 million) for ED visits were attributable to heat exposure in 2016 (126). In Germany, costs for heat-related hospital admissions were found to be six times higher during extreme heat periods compared to nonextreme periods (127). The economic impact in France was also substantial, with €25.5 billion attributed to selected health effects from extreme heat between 2005 and 2009 (128). These examples underscore the growing economic burden of extreme heat on public health systems worldwide, particularly in regions with high population densities and limited adaptive capacity.

The burden of disease from extreme heat is also significant. An Australian study covering the period 2003 to 2018 showed that high temperatures accounted for 2.7% of the observed burden of kidney diseases, resulting in an annual loss of 1,446.8 years of healthy life or 6.4 disability-adjusted life years (DALYs) per million people. Under a scenario of higher greenhouse gas emissions (e.g., representative concentration pathway 8.5), this burden is projected to quadruple by the 2050s (129). Similar trends have been observed in other regions, where climate projections indicate escalating health burdens if effective adaptation measures are not implemented.

Furthermore, extreme heat exacerbates occupational injuries and illnesses, increasing the overall disease burden and reducing productivity. An Australian study using workers compensation data from 2014 to 2019 found that heat contributed to approximately 42,884 DALYs lost due to occupational injuries, representing 2.3% of all occupational injury-related DALYs (130). The financial impact on healthcare costs associated with occupational injuries due to extreme heat amounted to approximately A$4.3 million annually (131).

Mitigation strategies to reduce the economic impact of extreme heat include implementing workplace-specific adaptation measures, such as shaded or climate-controlled environments, flexible work hours to avoid peak heat periods, and mandatory breaks for outdoor workers. Urban greening initiatives, such as increasing tree coverage and introducing green roofs, can also help lower urban temperatures, reducing both healthcare and energy costs. Policies promoting energy-efficient cooling technologies and ensuring equitable access to these technologies for low-income populations are critical to mitigating heat-related health and economic impacts.

## Heat Health Action Plans And Warning Systems: Development, Implementation, And Inequality

### Health Protection Responses to Extreme Heat

HHAPs that include HHWSs are considered effective adaptation responses to the health risks of extreme heat events (114). While HHAPs and HHWSs are increasingly implemented through multisectoral collaboration, they remain primarily grounded in public health. HHAPs are the frameworks used for planning and preparing for, mitigating, and responding to the health impacts of extreme heat. While they should be tailored to the local context and vulnerabilities, HHAPs commonly include the following: specification of roles and responsibilities; mechanisms for interagency coordination; capacity and capability building; public awareness raising and community outreach; health-protective actions to be taken by governments, services, healthcare professionals, and individuals; and monitoring and evaluation of impact (132, 133).

HHWSs are an essential component of HHAPs, providing information about the location, intensity, and duration of periods of extreme heat to prompt health-protective actions. HHWSs use climate and weather forecasts and established criteria to trigger the issuing of advisories and warnings before, during, and after an extreme heat event that have been preagreed to by health and meteorological authorities. These criteria usually include one or more indicators of population-level heat exposure (with or without humidity), as well as grade thresholds for expected health impacts and actions. However, there is enormous diversity across HHWSs in metrics and methods for determining appropriate trigger thresholds. A comparison of the capability of different approaches to identify days that were sufficiently hot to have significant health impacts found little agreement between approaches (synoptic, epidemiological, temperature humidity index, physiological classification) (134). The approach chosen is largely determined by the human and other resources available, including technical expertise and data availability (135).

### Genesis and Development of Heat Health Action Plans and Warning Systems

While extreme heat episodes earlier in the twentieth century were associated with significant health impacts, it was not until 1995 that the first formal “Hot Weather-Health Watch/Warning System” was established in the city of Philadelphia (136).

The imperative to develop systems to protect people from extreme heat gained ground following extreme heat events that led to substantial mortality, particularly in older and socially marginalized groups around the turn of the century. By 2005, 12 countries in Europe had HHWSs, and by 2018, 35 had HHAPs, although many of these were at the regional or city level, rather than the national level (137). In the United States, heat watch/warning systems based on the National Weather Service heat index metric were in place at the end of the 1990s, and a range of plans by state, local, tribal, and territorial governments have since evolved (136). Graded heat alerts are now issued by 122 weather forecast offices, considering a range of heat risk indicators (138).

Other countries have now also developed national-, regional-, or city-level HHWSs and HHAPs. However, coverage remains incomplete, especially in lower- and middle-income countries where populations are frequently the most exposed and vulnerable to the effects of extreme heat. In the report of the 2024 UN Secretary-General’s call to action on extreme heat, the World Health Organization (WHO) and World Meteorological Organization (WMO) estimate that the global scaling-up of HHWSs in 57 countries could potentially save over 98,000 lives per year (139).

With the greater experience in dealing with extreme heat events, the increasing accuracy of forecasting extreme heat events, the development and implementation of HHWSs and HHAPs in place, and the potential for some population-level acclimatization, responses continue to evolve, including revising thresholds for issuing warnings (140) and extending the season they are active (141). Emerging practices include the appointments of city-level “Chief Heat Officers,” naming and ranking heat waves (142), a greater emphasis on multiagency involvement (beyond health and weather services) (143), and novel technological approaches to communicating more personally relevant information (e.g., Google excessive heat warnings and apps).

### Equity Considerations

As noted, population coverage of HHWSs and HHAPs is incomplete and inequitable, meaning that large populations, particularly in low- and middle-income countries, do not have access to early warnings or interventions to protect their health. Barriers include the availability of financial resources, lack of meteorological and population health data, and capacity to design, implement, and evaluate these systems. While anyone can be vulnerable to extreme heat, some population groups are more vulnerable than others. Therefore, HHWSs and HHAPs should navigate different tiers of vulnerability, balancing the competing challenges of promoting protective actions for those most vulnerable while avoiding overwarning for the general population, which may cause messaging to be unheeded.

Most, if not all, HHWSs and HHAPs tailor responses toward those most at risk. Advice often emphasizes community solidarity, encouraging people to support more vulnerable members of their neighborhoods. Service responses include community outreach interventions, opening of shelters, distribution of drinking water, and specific responses for mass gatherings in the emergency phase. A few HHAPs include a longer-term focus on vulnerability and heat risk reduction, for instance, through social or built environment interventions. For example, England’s Heat Health Alert action cards include year-round actions for the government, commissioners, providers, and the non-governmental organizations (e.g., Red Cross or other local community initiatives) to increase heat resilience such as housing, infrastructure, and data sharing agreements (144).

However, simple communications and population-level interventions are unavoidably blunt and limited in their capability to deal with the complex and dynamic nature of vulnerability across diverse communities and geographies. This underscores the value of localized and community-based action. The specific challenges faced by workers in different industries may require more tailored and dedicated advice and responses than for the public in general.

### Humidity Considerations

Given the intricate relationship between high temperatures, humidity, and heat-related illness, it is important to consider the role of humidity in heat warning systems, as it may be key to improving public health responses to heat waves, especially for vulnerable populations. Currently, many HHWSs use only temperature-based metrics, such as maximum/minimum daily temperature (e.g., England) or an index such as the Excess Heat Factor (EHF), used in Australia. Some consider both temperature and humidity separately (e.g., France) or use a composite metric such as the heat index, apparent temperature, or the WBGT (e.g., United States, Canada, Germany, Japan). However, there are many different measures of air moisture content, and understanding the metric used is vital to interpreting the risk to health of advisories and warnings using these measures (88). Japan revised the country’s HHWS metric from air temperature to WBGT in 2021 (92) because of concerns about the health risks of humidity with heat, finding improved predictive capabilities for morbidity. Australia is exploring a heat index version of the EHF for very dry or very humid heat waves in tropical Australia (145). The European Centre for Medium-Range Weather Forecasts has produced preoperational forecasts of a heat health hazard index, which predicts the Universal Thermal Climate Index (UTCI) (146), a physiologically based thermal stress metric created to reflect average human physiological perception to the outdoor thermal environment (88). While this metric is more human-centric than others, its data inputs are quite demanding, so its use may be limited to countries that collect the necessary data and have the technical capacity to use it. No current HHWSs using this metric have been identified to date (135).

**Universal Thermal Climate Index (UTCI):** a bioclimatic index for describing the physiological comfort of the human body under specific meteorological conditions

### Effectiveness of Heat Health Action Plans and Warning Systems

The effectiveness of HHWSs and HHAPs is often evaluated annually at the end of the season or following years with extreme heat events. Evaluations of HHWSs and HHAPs are usually either of process (did they operate as intended) or of outcomes (did they reduce heat-related illness and death). Although many HHWSs and HHAPs have been implemented, the number of evaluations of outcomes in the peer-reviewed literature is limited, with most evaluations undertaken in high-income countries.

Evidence suggests that HHAPs and their accompanying HHWSs are effective in reducing morbidity and mortality during extreme heat events. However, the diverse nature of these programs and varying evaluation methods limit definitive conclusions on their overall benefit (88). Methodological challenges, among others, include attributing impact to the HHWSs/HHAPs, isolating the impact of particular components among the range of interventions deployed, and comparing across studies given the heterogeneity (147). One promising approach is evaluations that take advantage of isolated incremental changes, such as threshold changes, to understand the impact of particular components (140). Given the increasing frequency, intensity, and duration of heat waves, improving our understanding of effective interventions for different population groups in different settings is urgently needed.

## Strategies To Enhance Heat Resilience

While existing HHAPs and HHWSs have demonstrated effectiveness in mitigating the impacts of extreme heat events, significant limitations remain, as evidenced by the high incidence of heat-related outcomes. Equity in heat resilience, as well as the role of humidity in heat effects, requires a more comprehensive and in-depth investigation. The findings should be promptly and accurately applied to the development of HHAPs and HHWSs to maximize cost-effectiveness and social benefits.

To promote equity in the development of HHWSs, efforts should prioritize financial investment, capacity building (e.g., training local health professionals, enhancing institutional frameworks), and technological improvements (e.g., enhancing data accessibility, strengthening heat-health relationship assessments, improving weather forecasting), particularly in low-income regions. To enhance the accuracy and effectiveness of HHWSs, efforts should focus on conducting regular evaluations, implementing evidence-based updates, and prioritizing the most vulnerable populations in warning responses. A comprehensive monitoring and evaluation framework should be established to promptly collect and analyze data, ensuring the effectiveness of interventions and the timely updating of strategies. Updates to HHWSs should focus on incorporating new data from ongoing research and integrating advanced technological tools to enhance accuracy and effectiveness, such as artificial intelligence and big data analysis.

**Figure 4:**
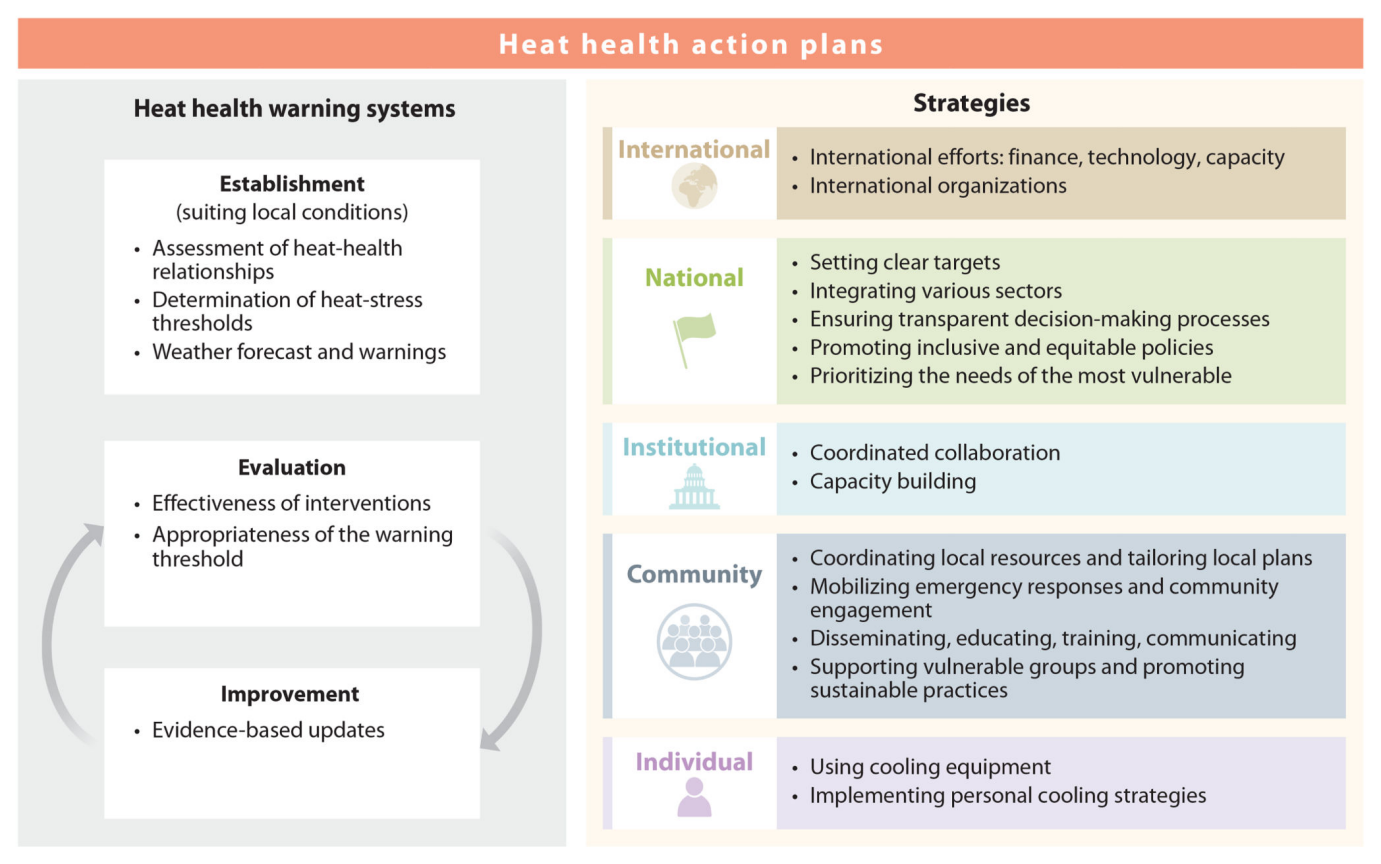
Proposed strategies to enhance heat resilience. This figure outlines a multilevel framework for heat health action plans. On the left, heat health warning systems are structured around three core components: establishment, evaluation, and improvement. On the right, a hierarchical strategy is proposed across international, national, institutional, community, and individual levels to enhance heat resilience.

To enhance heat resilience, we propose implementing HHAPs across the following hierarchical scales: international, national and institutional, community, and individual levels (Figure 4).

### International Level

At the international level, coordinated efforts are critical for advancing climate equity and sustainable development, accelerating mitigation and adaptation efforts and thereby making global heat resilience more attainable. The primary focus of international efforts is on finance investment, technology innovation, and capacity building, while addressing the barriers posed by regional disparities and resilience gaps (148). International organizations (e.g., WHO, WMO) play a key role in coordinating international collaboration, guiding policy development, fostering global consensus, promoting technical and financial support, facilitating knowledge dissemination, and supporting capacity building to strengthen global resilience to heat.

### National and Institutional Levels

At the national level, heat resilience actions are driven by political commitment, institutional frameworks, and effective governance. National governments play a critical role in setting clear targets, integrating various sectors, ensuring transparent decision-making processes, and promoting inclusive and equitable policies (148). Prioritizing the needs of the most vulnerable is crucial to ensure that adaptation and mitigation strategies are effective and equitable. At the institutional level, coordinated collaboration and capacity building are essential for the effectiveness of heat resilience strategies. Institutions should focus not only on reducing emissions but also on implementing adaptation measures to mitigate and adapt to the heat effects. Institutions (e.g., health sectors, meteorological agencies) must work together across sectors to monitor heat risks, develop accurate forecasts, conduct collaborative research, and prepare emergency responses, with accurate warning information promptly communicated to the public and communities to ensure timely and effective interventions (148).

### Community Level

Communities are crucial in responding to heat, ensuring that action plans are tailored to the local context, including strengths, constraints, and vulnerabilities (149). Communities play a vital role in tailoring heat action plans to local needs, coordinating local resources, managing emergency responses, mobilizing resident and institution engagement, disseminating heat health knowledge, organizing education and training, facilitating communication with local stakeholders, supporting vulnerable groups, and promoting sustainable practices. For example, communities are recommended to establish public shelters (e.g., libraries, public pools, water refilling stations), implement home improvement services, raise public awareness through education and training, and promote urban greening efforts (150). Evaluating the effectiveness of these interventions is also essential to ensure continuous improvement and adaptation to the community’s evolving needs (149).

### Individual Level

At the individual level, awareness of heat risks and implementation of effective actions are critical yet challenging (4). Individual actions can include using cooling equipment and implementing personal cooling strategies. Using cooling equipment typically incurs high costs, such as air conditioners and evaporative coolers (133). In contrast, personal cooling strategies usually involve more accessible and lower-cost methods, such as reducing higher-calorie diets, controlling physical activity, using fans, cooling the body (e.g., self-dousing, applying ice towels, consuming cold water and foods), and optimizing clothing choices (133). It is important to note that the effectiveness of these personal cooling strategies might be influenced by humidity (88). For example, in extreme heat and high humidity environments, fans are less effective for older adults (*>*65 years) with reduced sweating capacity (151); while in extreme heat and dry environments, fans can be detrimental as they may exacerbate physiological heat strain (133). A key challenge is the limited feedback from the core body temperature to provide adequate warning of individual heat-related trouble (4). This challenge is anticipated to be addressed through heat health education, ambient environment surveillance, and individual temperature monitoring (e.g., wearable thermometry devices).

## Summary

Extreme heat events increasingly pose significant global health risks, especially in vulnerable and underresourced regions. Despite advancements in research and interventions, numerous challenges persist, such as inconsistent data, the lack of a unified definition for heat waves, and a limited understanding of the combined effects of heat and humidity. Addressing these gaps necessitates coordinated, long-term efforts. These efforts should include improved data collection and consistency, the strengthening of HHWSs and HHAPs with consistent evaluations of their effectiveness and accessibility, and enhanced global collaboration to more efficiently mitigate the health hazards associated with extreme heat events.

## Supplementary Material

Supplementary Information

## Summary Points

1. Extreme heat events are intensifying globally, with particularly severe health impacts in underresourced regions.
2. Extreme heat substantially affects human health, disproportionately impacting vulnerable populations.
3. Epidemiological evidence on the role of humidity in modifying the effects of extreme heat remains inconsistent.
4. Extreme heat strains energy production, increases consumption, and leads to economic loss.
5. While existing heat health action plans and warning systems have made progress, further efforts are necessary to enhance the effectiveness.
6. Mitigating heat health risks requires a long-term commitment and coordinated efforts, involving top-down governance and bottom-up engagement.

## Future Issues

1. Many regions, especially the Middle East, lack reliable epidemiological and weather data. Global efforts are needed to support better data collection in these areas. Collaborations with local experts in underrepresented regions such as Africa, the Asian subcontinent, and Latin America could help address critical data gaps and enhance the effectiveness of regional heat-related research and interventions.
2. The absence of a unified definition of heat waves highlights the need for global collaboration to develop standardized criteria, enabling more effective comparisons and interventions across regions.
3. Despite extensive research on the health effects of extreme heat, the compound risks of extreme heat combined with humidity remain insufficiently explored. Further research is essential to fully understand these combined impacts on health.
4. Although the biological mechanisms have been somewhat elucidated, further explorations from the mechanisms of biology, psychology, ecology, epidemiology, and socioeconomics are warranted.
5. Regional disparities driven by socioeconomic factors affect the development and implementation of heat health warning systems (HHWSs) and heat health action plans (HHAPs). Future efforts should prioritize ensuring the equity of HHWSs and HHAPs, with a particular focus on low-income countries.
6. The implementation, effectiveness, and cost-efficiency of HHWSs and HHAPs remain largely unassessed. Comprehensive evaluations are recommended to inform evidence-based updates and improvements, particularly in regions such as Africa and Latin America where such assessments are scarce.
