# Supplementary material for: Lethal Heat and Humidity Events
Source: Annu Rev Environ Resour. Author manuscript; Available in PMC 2025 Oct 13. (PMC7618245; doi:10.1146/annurev-environ-111523-102139)
Supplement: Supplementary Information [file EMS209521-supplement-Supplementary_Information.pdf]

Downloaded from www.annualreviews.org. Guest (guest) IP: 193.62.199.199 On: Fri, 17 Oct 2025 11:54:27

Supplemental Methods

Meteorological data

Hourly meteorological data were obtained from the fifth-generation European Centre for Medium-Range Weather Forecasts (ECMWF) Reanalysis (ERA5) (1), at a spatial resolution of 0.25° × 0.25°. To align with local time zones, these hourly data were aggregated to calculate daily meteorological parameters. Hourly data were aggregated to calculate daily meteorological parameters, aligning with local time zones. The parameters included: daily mean 2 m ambient temperature (temperature measured at 2 meters above the Earth's surface), daily mean 2 m dew point temperature, daily mean eastward and northward wind components at 10 m, and daily mean surface downward short-wave radiation. Daily mean relative humidity (RH) was calculated using the daily mean ambient temperature and dew point temperature, with the “humidity” package in R (2).

Heatwave definition

A heatwave in each grid cell was defined as a period of at least two consecutive days where daily temperature metrics, specifically daily mean ambient temperature and Wet Bulb Globe Temperature (WBGT), exceeded the 95th percentile of the year-round frequency distribution for that cell (3).

WBGT measures heat stress in direct sunlight, considering temperature, humidity, wind speed, solar radiation, and sun angle. Various methods exist for calculating WBGT. Here we have used the physics-based model developed by Liljegren et al. (4), extensively used and regarded as one of the best (5). This model applies mass and energy balance principles to simulate heat stress conditions.

In this study, WBGT was calculated using Liljegren’s model, represented by the following equation:

$$WBGT = 0.7T_{nwb} + 0.2T_g + 0.1T_a, \quad (1)$$

where T_{nwb} is the natural wet-bulb temperature (°C); T_g is globe temperature (°C); and T_a is the ambient air temperature (°C), derived directly as the 2 m temperature from the ERA5 dataset. The natural wet-bulb temperature and globe temperature were calculated iteratively using inputs such as dew point temperature, relative humidity, wind speed, and intercepted solar radiation. We used the Liljegren's algorithm implementation available in the 'HeatStrees' package in R (6, 7). The Sen's slope estimator was calculated using the "Trend" package in R to quantify the decadal change in heatwave days. A positive Sen's slope indicates an increasing trend, while a negative value reflects a decreasing trend. All the software used here was free software, to comply with the requirements of code availability and reproducibility (8).

Downloaded from www.annualreviews.org. Guest (guest) IP: 193.62.199.199 On: Fri, 17 Oct 2025 11:54:27

Supplemental Table 1. Health effect of extreme heat and humidity [a]

Category of Health condition	Representative conditions affected by heat	Examples of findings about the health effect of heat	Examples of findings about the modification effect of humidity
Heat-related illness	Heat rash, heat exhaustion, heat stroke	A meta-analysis combining data from 30 studies found that for a 1°C increase in temperature above the study-specific baseline, morbidity and mortality from direct heat-related illness rose by 18% and 35% (RR=1.35, 95%CI=[1.29–1.41]), respectively (9). The most significant increase in morbidity was reported for direct heat illness (RR=1.45, 95%CI=[1.38–1.53]), compared to dehydration (RR=1.02, 95%CI=[1.02–1.03]). The risk was particularly elevated for individuals over 65 years (RR=1.25, 95%CI=[1.20–1.30]) and those residing in subtropical climates (RR=1.25, 95%CI=[1.21–1.29]) (9).	No direct evidence.
Cardiorespiratory disease	Ischemic heart disease (IHD) including acute IHD and chronic IHD, myocardial infarction, stroke, heart failure, and chronic obstructive pulmonary disease	A meta-analysis of 54 studies showed that the relative risks of heatwaves on cardiovascular and respiratory mortality were 1.15 (95% CI:1.09, 1.21) and 1.18 (95%CI:1.09, 1.28), respectively(10).	• A time-series study conducted in 11 cities in China found that the impact of temperature on cardiovascular mortality was higher at high humidity(11).<br>• However, another time-series study conducted in 353 locations in China found that the mortality risks for cardiovascular disease (14.30%, 95%CI: 12.01%, 16.64%) and respiratory disease (12.99%, 95%CI: 10.21%, 15.85%) was higher during dry-hot events compared to wet-hot events(12).

Downloaded from www.annualreviews.org. Guest (guest) IP: 193.62.199.199 On: Fri, 17 Oct 2025 11:

Downloaded from www.annualreviews.org. Guest (guest) IP: 193.62.199.199 On: Fri, 17 Oct 2025 11:5

			• A study of 48 provinces in Spain found that humidity significantly modified the association between ambient heat and acute bronchitis and bronchiolitis admission, with rhigher risks during dry days (13).<br>• A systematic review showed that low-intensity heatwaves can increase all-cause, particularly cardiovascular-, respiratory- and diabetes-related mortality, in areas experiencing hot, humid summers (14).
Infectious disease	Infectious and parasitic diseases, respiratory infectious disease, intestinal infectious enteritis, dengue	• A meta-analysis of 57 studies showed increased relative risks of incidence were 1.05 (95% CI: 1.04,1.07) for salmonellosis, 1.07 (95% CI: 1.04,1.10) for shigellosis, 1.02 (95% CI: 1.01,1.04) for campylobacteriosis, 1.05 (95% CI: 1.04-1.07) for cholera, 1.04 (95% CI: 1.01-1.07) for Escherichia coli enteritis, and 1.15 (95% CI: 1.07,1·24) for typhoid; reduced risks were 0.96 (95%CI: 0.90,1.02) for rotaviral enteritis and 0.89 (0.81,0·99) for noroviral enteritis per 1°C temperature increase (15).<br>• A meta-analysis of 54 studies showed a 13% increase in risk of dengue infection (RR =1.13; 95%CI: 1.11-1.16) for each 1 °C increase in temperature(16).	No direct evidence.
Kidney disease	Acute renal injury, renal failure, nephrolithiasis, urolithiasis, urinary tract infections, chronic kidney diseases	• A meta-analysis of 82 studies showed relative risks were 1.010 (95%CI: 1.009,1.011) for kidney-related morbidity and 1.031 (95%CI: 1.018,1.045) for kidney-related mortality for each 1°C increase in temperature (17).	No direct evidence.

Mental and behavioural disorders	Schizophrenia, mood disorders, organic mental disorders (including dementia), neurotic disorders (such as anxiety and depression), psychoactive substance use-related disorders	• A meta-analysis of 7 studies showed that the risk of MBD morbidity increased during extreme heat with a pooled RR of 1.05 (95%CI: 1.02-1.08) compared with non-extreme-heat periods (18).<br>• A meta-analysis of 3 studies reported an 9.7% increase in the risk of hospital attendance or admissions for mental illness during extreme heat compared with non-extreme-heat periods (19).<br>• A meta-analysis showed that the relative risks were 1.031 (95% CI: 1.011–1.052), 5 studies) for MBD mortality and 1.007 (95% CI: 1.005–1.009, 12 studies) for 1 ºC increase in temperature (20).	No direct evidence.
Metabolic disease	Diabetes, dyslipidemia, hypertension, hyperglycemia, hyperuricemia.	A study of 48 provinces in Spain found that the strongest impact of extreme heat was observed for metabolic disease (RR = 1.98, 95%CI:1.77, 2.21) (13).	• A systematic review showed that low-intensity heatwaves can increase all-cause, particularly cardiovascular-, respiratory- and diabetes-related mortality, in areas experiencing hot, humid summers (14).<br>• A study in Wuhan found that ultra-high relative humidity and temperature increased the mortality rate of metabolic disease (21).
Pregnancy and birth outcomes	Gestational diabetes mellitus, hypertensive disorders of pregnancy, preterm birth, low birth weight, small or large for gestational age, stillbirth, infant mortality, congenital heart defects	A meta-analysis of 6 studies showed that the relative risks were 1.05 (95%CI: 1.03, 1.07, 6 studies) for preterm birth and 1.05 (95%CI: 1.01,1.08, 8 studies) for stillbirth for a 1°C increase in temperature (22).	No direct evidence.

a: This list is not comprehensiv

Downloaded from www.annualreviews.org. Guest (guest) IP: 193.62.199.199 On: Fri, 17 Oct 2025 11:[illegible]

Reference

1. Hersbach H, Bell, B., Berrisford, P., Biavati, G., Horányi, A., Muñoz Sabater, J., Nicolas, J., Peubey, C., Radu, R., Rozum, I., Schepers, D., Simmons, A., Soci, C., Dee, D., Thépaut, J-N. 2023. ERA5 hourly data on single levels from 1940 to present.: Copernicus Climate Change Service (C3S) Climate Data Store (CDS)
2. Cai J. 2018. humidity: Calculate Water Vapor Measures from Temperature and Dew Point. R package version 0.1.4.
3. Guo Y, Gasparrini A, Li S, Sera F, Vicedo-Cabrera AM, et al. 2018. Quantifying excess deaths related to heatwaves under climate change scenarios: A multicountry time series modelling study. *PLoS medicine* 15: e1002629
4. Liljegren JC, Carhart RA, Lawday P, Tschopp S, Sharp R. 2008. Modeling the wet bulb globe temperature using standard meteorological measurements. *J Occup Environ Hyg* 5: 645-55
5. Kong Q, Huber M. 2022. Explicit Calculations of Wet-Bulb Globe Temperature Compared With Approximations and Why It Matters for Labor Productivity. *Earth's Future* 10: e2021EF002334
6. Casanueva A, Kotlarski S, Fischer AM, Flouris AD, Kjellstrom T, et al. 2020. Escalating environmental summer heat exposure—a future threat for the European workforce. *Regional Environmental Change* 20: 40
7. Casanueva A, Kotlarski S, Herrera S, Fischer AM, Kjellstrom T, Schwierz C. 2019. Climate projections of a multivariate heat stress index: the role of downscaling and bias correction. *Geosci. Model Dev.* 12: 3419-38
8. Añel JA. 2011. The importance of reviewing the code. *Commun. ACM* 54: 40–41
9. Faurie C, Varghese BM, Liu J, Bi P. 2022. Association between high temperature and heatwaves with heat-related illnesses: A systematic review and meta-analysis. *Sci Total Environ* 852: 158332
10. Cheng J, Xu Z, Bambrick H, Prescott V, Wang N, et al. 2019. Cardiorespiratory effects of heatwaves: A systematic review and meta-analysis of global epidemiological evidence. *Environ Res* 177: 108610
11. Zeng J, Zhang X, Yang J, Bao J, Xiang H, et al. 2017. Humidity May Modify the Relationship between Temperature and Cardiovascular Mortality in Zhejiang Province, China. *Int J Environ Res Public Health* 14: 1383
12. Fang W, Li Z, Gao J, Meng R, He G, et al. 2023. The joint and interaction effect of high temperature and humidity on mortality in China. *Environment International* 171: 107669
13. Achebak H, Rey G, Chen ZY, Lloyd SJ, Quijal-Zamorano M, et al. 2024. Heat Exposure and Cause-Specific Hospital Admissions in Spain: A Nationwide Cross-Sectional Study. *Environ Health Perspect* 132: 57009
14. Strathearn M, Osborne NJ, Selvey LA. 2022. Impact of low-intensity heat events on mortality and morbidity in regions with hot, humid summers: a scoping literature review. *Int J Biometeorol* 66: 1013-29
15. Chua PLC, Ng CFS, Tobias A, Seposo XT, Hashizume M. 2022. Associations between ambient temperature and enteric infections by pathogen: a systematic review and meta-analysis. *The Lancet Planetary Health* 6: e202-e18
16. Damtew YT, Tong M, Varghese BM, Anikeeva O, Hansen A, et al. 2023. Effects of high temperatures and heatwaves on dengue fever: a systematic review and meta-analysis. *EBioMedicine* 91: 104582
17. Liu J, Varghese BM, Hansen A, Borg MA, Zhang Y, et al. 2021. Hot weather as a risk factor for kidney disease outcomes: A systematic review and meta-analysis of epidemiological evidence. *Science of The Total Environment* 801: 149806
18. Li D, Zhang Y, Li X, Zhang K, Lu Y, Brown RD. 2023. Climatic and meteorological exposure and mental and behavioral health: A systematic review and meta-analysis. *Science of the Total Environment* 892: 164435

Downloaded from www.annualreviews.org. Guest (guest) IP: 193.62.199.199 On: Fri, 17 Oct 2025 11:54:27

19. Thompson R, Lawrance EL, Roberts LF, Grailey K, Ashrafian H, et al. 2023. Ambient temperature and mental health: a systematic review and meta-analysis. *The Lancet Planetary Health* 7: e580-e89
20. Liu J, Varghese BM, Hansen A, Xiang J, Zhang Y, et al. 2021. Is there an association between hot weather and poor mental health outcomes? A systematic review and meta-analysis. *Environment international* 153: 106533
21. Zhang T, Ni M, Jia J, Deng Y, Sun X, et al. 2023. Research on the relationship between common metabolic syndrome and meteorological factors in Wuhu, a subtropical humid city of China. *BMC Public Health* 23: 2363
22. Chersich MF, Pham MD, Areal A, Haghighi MM, Manyuchi A, et al. 2020. Associations between high temperatures in pregnancy and risk of preterm birth, low birth weight, and stillbirths: systematic review and meta-analysis. *BMJ* 371: m3811

Downloaded from www.annualreviews.org. Guest (guest) IP: 193.62.199.199 On: Fri, 17 Oct 2025 11:54:27
